# Mapping onto Eq-5 D for patients in poor health

**DOI:** 10.1186/1477-7525-8-141

**Published:** 2010-11-26

**Authors:** Matthijs M Versteegh, Donna Rowen, John E Brazier, Elly A Stolk

**Affiliations:** 1 iMTA/iBMG, Erasmus University of Rotterdam, PO Box 1738, 3000 DR Rotterdam, The Netherlands; 2 School of Health and Related Research, University of Sheffield, Regent Court, 30 Regent Street, Sheffield S1 4DA, UK

## Abstract

**Background:**

An increasing amount of studies report mapping algorithms which predict EQ-5 D utility values using disease specific non-preference-based measures. Yet many mapping algorithms have been found to systematically overpredict EQ-5 D utility values for patients in poor health. Currently there are no guidelines on how to deal with this problem. This paper is concerned with the question of why overestimation of EQ-5 D utility values occurs for patients in poor health, and explores possible solutions.

**Method:**

Three existing datasets are used to estimate mapping algorithms and assess existing mapping algorithms from the literature mapping the cancer-specific EORTC-QLQ C-30 and the arthritis-specific Health Assessment Questionnaire (HAQ) onto the EQ-5 D. Separate mapping algorithms are estimated for poor health states. Poor health states are defined using a cut-off point for QLQ-C30 and HAQ, which is determined using association with EQ-5 D values.

**Results:**

All mapping algorithms suffer from overprediction of utility values for patients in poor health. The large decrement of reporting 'extreme problems' in the EQ-5 D tariff, few observations with the most severe level in any EQ-5 D dimension and many observations at the least severe level in any EQ-5 D dimension led to a bimodal distribution of EQ-5 D index values, which is related to the overprediction of utility values for patients in poor health. Separate algorithms are here proposed to predict utility values for patients in poor health, where these are selected using cut-off points for HAQ-DI (> 2.0) and QLQ C-30 (< 45 average of QLQ C-30 functioning scales). The QLQ-C30 separate algorithm performed better than existing mapping algorithms for predicting utility values for patients in poor health, but still did not accurately predict mean utility values. A HAQ separate algorithm could not be estimated due to data restrictions.

**Conclusion:**

Mapping algorithms overpredict utility values for patients in poor health but are used in cost-effectiveness analyses nonetheless. Guidelines can be developed on when the use of a mapping algorithms is inappropriate, for instance through the identification of cut-off points. Cut-off points on a disease specific questionnaire can be identified through association with the causes of overprediction. The cut-off points found in this study represent severely impaired health. Specifying a separate mapping algorithm to predict utility values for individuals in poor health greatly reduces overprediction, but does not fully solve the problem.

## Background

In recent years there has been an increasing amount of publications concerned with 'mapping' condition specific measures on EQ-5 D to estimate EQ-5 D utility values. Mapped EQ-5 D utility values are accepted as evidence in cost-utility analyses by reimbursement agencies such as the National Institute of Health and Clinical Excellence (NICE) [1] (see § 5.4.6) but suffer from non-trivial problems like the overprediction of utility values for patients in poor health. A mapping algorithm can be estimated by regressing a non-preference-based measure onto a preference-based measure on a dataset external to your study dataset [2]. The resulting mapping equation is used to estimate the utility values of the preferenced-based measure in the study dataset where such a measure is absent. Criteria for the quality of a mapping algorithm do not currently exist although it is well known that utilities estimated by mapping algorithms typically have larger errors for lower utility values [2] and mapped EQ-5 D utilities show a systematic overprediction of utility values for patients in poor health [3]. For instance, a study mapping SF-12 on EQ-5 D report predicted values under 0.5 to be notably higher than observed values, for both 2^nd ^and 4^th ^order models [4]. Another study, mapping the modified Rankin scale measurement, which assesses disability after stroke, on EQ-5 D reports decreased accuracy for patients in poor health and significant overprediction of low values [5]. While it is unlikely for such overprediction to be a problem in all samples, given that many studies have reasonably high mean EQ-5 D values [6], it is likely to occur in patient (sub) samples containing a significant proportion of individuals in poor health. The current study explores whether the causes of overprediction of utility values for patients in poor health found in the literature can inform a method to minimize that overprediction. The proposed solution involves the use of a different algorithm for patients in poor health, where health status is determined using available information from a condition-specific non-preference-based measure.

There are several causes for the overprediction of low utility values. First, the non-preference based measure may have different severity content than the preference-based measure. For instance, the lowest possible range of scores on the Health Assessment Questionnaire Disability Index (HAQ-DI) is between 2.5 and 3.0 which is not necessarily associated with the lowest value of -.59 on the EQ-5 D, but with a value near .1 [7], as the HAQ measures different dimensions of health [8]. Adding additional covariates to the mapping functions, like clinical variables or dimension scores of other questionnaires may overcome this problem, but this limits the use of the function to datasets that hold all those variables.

Second, in many clinical studies, health states are not normally distributed: most patients typically experience mild to moderate health problems and few experience severe problems [6,8,9]. Progression from moderate to poor states, for instance moving from 'some problems with washing or dressing myself' to 'unable to wash or dress myself', results in a steep drop in utilities. This 'drop' may not be adequately predicted in a linear model which is powered on the large group of patients which reports mild to moderate health problems. This has led to the suggestion that using Ordinary Least Squares regression on the entire sample, which is more accurate for mean values than for extremes, may contribute to the problem of overprediction [2]. Specifying other models may lead to better predictions, but will rarely overcome overprediction.

Alternatively, one option is to specify a separate mapping function for patients in poor health whose utility values are overpredicted. Such an approach would require a method to identify the 'poor health' population. A study, mapping SF-36 onto EQ-5 D, reported overprediction of utility values for poorer health states (EQ-5 D index values < 0.5) for existing algorithms from the literature and algorithms estimated in the study [3]. The study hypothesized that this may be observed because more severe health states (utility value <0.5) have at least one of five EQ-5 D health dimensions at the most severe level causing the aforementioned steep decline in utility values. Further support for this hypothesis is that in many patient populations a 'bimodal distribution' of EQ-5 D utility values is observed. Bimodal distribution refers to the observation of high (> 0.5) mean utility values for EQ-5 D states with no dimensions at the most severe level and low (< 0.5) mean utility values for EQ-5 D states with one or more dimensions at the most severe level. This bimodal distribution has a 'gap' in the distribution of EQ-5 D utility values around the .5 value [9]. This observation is limited to EQ-5 D, as prediction errors are also increased for patients in poor health when mapping to SF-6 D [10], but no systematic overprediction is present.

This suggests that the alternative mapping function ought to be estimated on the lower part of the bimodal distribution of EQ-5 D values. However, as the EQ-5 D is absent by definition if a mapping algorithm is applied, it is difficult to assess which predicted values are overpredicted. It is plausible that values can be identified on the condition-specific instrument that are associated with the lower part of the EQ-5 D utility distribution, which represents 'poor health'. To this purpose mapping algorithms and datasets for three condition-specific measures, the arthritis Health Assessment Questionnaire (HAQ) and the cancer EORTC's Quality of Life Questionnaire C-30 (version 2) are investigated. When available mapping algorithms systematically overpredict utility values for patients in poor health, it is explored whether it is possible to identify the 'poor health' population by the health status reported on the condition specific measure. If so, we estimate a separate mapping algorithm for use in patients in poor health.

## Method

Existing and new mapping algorithms will be applied to one sample of patients with arthritis [11] (arthritis sample) and two samples of patients with cancer: patients with Multiple Myeloma (MH sample) and patients with Non-Hodgkin's Lymphoma (NH sample) [12,13]. A short description of the population characteristics of the samples (pooled data for 8 follow-up time points of QLQ-C30, baseline of HAQ) on which the algorithms are run is presented in Table 1. Thus all work presented in this paper is performed using these datasets, limiting generalizability to different types of cancer.

**Table 1:** Patient characteristics

EQ-5D		N	Mean		% at level 1/2/3*
*Multiple Myeloma population (pooled)*					
			
Age (range)		652	54 (37 - 65)		
EQ-5D	Mobility				56,7/41,4/1,9
	Self-care				85,8/12,8/1,4
	Usual activities				30,1/51,1/18,8
	Pain/Discomfort				39,6/59/1,4
	Depression/Anxiety				69,4/29,6/1,0
	EQ-5 D utility (UK tariff)		,69 (-,32 - 1)		
Male/Female		381/252			
Follow-up series		t = 0, 1, 2, 3, 4, 5, 6, 7			
*Non-Hodgkin population (pooled)*					
			
Age (range)		789	72 (65 - 84)		
EQ-5D	Mobility				48/47,3/4,7
	Self-care				81,4/13,9/4,7
	Usual activities				38,1/43,3/18,6
	Pain/Discomfort				52,2/42,9/4,9
	Depression/Anxiety				70/29/1,0
	EQ-5 D utility (UK tariff)		,68 (-,59 - 1)		
Male/Female		480/351			
Follow-up series		t = 0, 1, 2, 3, 4, 5, 6, 7, 8			
*Arthritis population*					
			
Age (range)		457	50 (16 - 88)		
EQ-5D	Mobility				58,5/41,5/0
	Self-care				75,3/24,3/,4
	Usual activities				37,1/58,2/4,7
	Pain/Discomfort				9/77,4/13,6
	Depression/Anxiety				70,7/27,1/2,2
	EQ5 D utility (UK tariff)		,62 (-,24 - 1)		
Male/Female		133/333			
Follow-up series		t = 0			
**Condition specific instruments**					

EORTC QLQ-C30 (Sum scores)				HAQ (Domain scores)	
			
	*MM population mean (SD)*	*NH Population mean (SD)*		*Arthritis population mean (SD)*	
	
Physical functioning	64 (24,6)		57,3 (26,8)	Dressing & Grooming	0,58 (,71)
Role functioning	59,5 (28,9)		57,4 (31,5)	Arising	0,65 (,73)
Emotional functioning	82,8 (18,9)		81,3 (20,6)	Eating	0,75 (,82)
Cognitive functioning	82 (20,8)		81,9 (23,7)	Walking	0,54 (,78)
Social functioning	76,2 (25,8)		75,7 (28,6)	Hygiene	0,64 (,81)
Global health	68,7 (18,0)		62 (21,7)	Reach	0,64 (,75)
Fatigue	35,7 (25,0)		44,7 (44,7)	Grip	0,78 (,85)
Nausea/Vomiting	6,1 (14,3)		8 (16,9)	Activities	0,94 (,88)
Pain	25,2 (24,7)		18,7 (26,2)		
Dyspnoea	16,1 (24,9)		24,8 (28,9)		
Sleep	21,1 (27,3)		28,7 (31,8)		
Appetite	16 (27,2)		21,9 (32,6)		
Constipation	4 (15,4)		11,8 (22,8)		
Diarrhea	8,3 (18,7)		7 (18,5)		
Financial difficulties	12,5 (23,0)		6,3 (16,9)		

* EQ-5D: 1/2/3 = no problems/moderate problems/severe problems

### Instruments

The EuroQol EQ-5 D is a generic preference-based measure of health related quality of life. It classifies health states on five dimensions (mobility; self-care; usual activities; pain/discomfort and anxiety/depression) with three severity levels each: level one represents no problems; level two represents some problems; and level three represents extreme problems. The classification system defines 243 unique health states which are given a utility score using an existing tariff. The EQ-5 D tariff represents the preferences of the general public as elicited using time trade-off, and differs per country. Here the UK tariff [14] and Dutch tariff [15] are used.

The EORTC QLQ-C30 (version two) is a cancer specific questionnaire which consists of 30 items across 6 functioning scales (physical, role, cognitive, emotional, social, global quality of life) and 9 symptom scales (fatigue, nausea and vomiting, pain, dyspnoea, sleep disturbance, appetite loss, constipation, diarrhoea, financial impact). High scores on the functioning and global health status scales reflect good quality of life, while high scores on the symptom scales represent a high level of symptoms [16].

The Health Assessment Questionnaire (HAQ) was first developed for use in patients in rheumatology. The most widely used version of the HAQ assesses the functional ability of patients using 20 items across eight domains (dressing, arising, eating, walking, hygiene, reach, grip and usual activities) [17]. Questions are scored on a four level disability scale from zero to three, where three represents the highest degree of disability. Scores for the eight domains are adjusted for the use of aids or devices and averaged into an overall disability index value, HAQ-DI (Health Assessment Questionnaire Disability Index), with a range from zero to three and adjacent steps of 0.125 (e.g. 0, 0.125, 0.250), which represents the extent of functional ability of the patient. A value between one and two represents moderate to severe disability [18].

### Algorithms

Algorithms are taken from the literature and predict EQ-5 D index values from either the QLQ-C30 (version 2) or the HAQ. All algorithms have been tested on another dataset with the exception of one HAQ model that was developed for this article, from now on referred to as a test model.

The original articles in which the algorithms were presented labelled them as suitable for estimating utility values [8,19,20]. Details of the algorithms are presented in Table 2. All models were developed using ordinary least squares regression. The HAQ algorithm developed and tested by Bansback et al. [19] was estimated on patient samples from Canada (N = 319) and the United Kingdom (N = 151) who were clinically diagnosed with rheumatoid arthritis (RA). The algorithm computes EQ-5 D utility values based on the UK tariff. We estimated one additional HAQ algorithm, the test model, for this article based on a larger group of patients than was used for the published algorithm, as this sample holds more patients in severe conditions [8]. The test model was developed using the Rotterdam Early Arthritis Cohort with 493 patients with and without clinically diagnosed RA recruited from the Erasmus Medical Centre in the Netherlands. It is not recommended for use as not all patients are clinically diagnosed with RA. A tested HAQ model that predicts Dutch utilities is presented elsewhere [8]. The QLQ-C30 algorithm by McKenzie & Van der Pol [20] was developed on a sample of 199 patients with inoperable esophageal cancer. The algorithm computes EQ-5 D utility values based on the UK tariff. The QLQ-C30 algorithm by Versteegh et al. [8] was developed and tested on pooled data from two clinical trials for patients with multiple myeloma (pooled N = 723) and patients with aggressive non-Hodgkin's lymphoma (pooled N = 789). It computes EQ-5 D utility values based on the Dutch tariff.

**Table 2:** Mapping algorithm specifications

Measure		Algorithms
HAQ	Bansback (2006)^1^	EQ-5 D index (UK tariff) = .80 + (h1_2*-.15) + (h4_1*-.08) + (h4_2*-.12) + (h4_3* -.59) + (h6*-.15) + (h7_1*-.04) + (h7_2*-.08) + (h8*-.10) + (h9*.12) + (h13* -.14) + (h16*.07) + (h23*-.05) + (h24_1*-.05) + (h24_2*-.11) + (h26_2*-.14) + (h26_3*-.13) + (h27_2*-.08) + (h27_3*-.20) + (h30_1*-.05) + (h31_1*-.07) + (h31_2*-.08) + (h32*.09)
	Test model2*	EQ-5 D index (Dutch tariff) = 0,858 + (haq1* -0,027) + (haq2*-0,035) + (haq3*-0,025) + (haq4*-0,033) + (haq5*-0,001) + (haq6*-0,035) + (haq7*-0,031) + (haq8*-0,057)
QLQ-C30	McKenzie (2009)^3^	EQ-5 D index (UK tariff) = .2376 + (ql*.0016) + (pf*.0004) + (rf*.0022) + (ef*.0028) + (cf*.0009) + (sf*.0002) + (fa*-.0021) + (nv*.0005) + (pa*-.0024) + (dysp*.0004) + (sleep*.00004) + (eat*.0003) + (obsti*.0001) + (diarr*-.0003) + (finan*-.0006).
	Versteegh (in press)^4^	EQ-5 D index (Dutch tariff) = 0.985 = (1*-.037) + (2*-.025) + (3*-.059) + (4*-.033) + (5*-.134) + (6_level2*-.033) + (6_level3*-.067) + (6_level4*-.180) + (7_level2*-.013) + (7_level3*-.037) + (7_level4*-.012) + (9_level2*-.065) + (9_level3*-.053) + (9_level4*-.189) + (16_level2*-.038) + (16_level3*-.045) + (16_level4*-.126) + (23_level2*-.028) + (23_level3*-.049) + (23_level4*-.456) + (24_level2*-.053) + (24_level3*-.140) + (24_level4*-.232) + (27_level2*-.027) + (27_level3*-.091) + (27_level4*-.110).

^1 ^HAQ items as dummy variables: h1 = dressing & grooming; h4 = arising; h6-7 = eating; h8-9 = walking; h13-16 = aids or devices; h23-24 = hygiene; h26 = reach; h27-28 = grip; h30-32 = activities. (e.g. h1_2 = haq item one, answer level two).

^2 ^HAQ sum scores: haq1 = dressing & grooming; haq2 = arising; haq3 = eating; haq4 = walking; haq5 = hygiene; haq6 = reach; haq 7 = grip; haq8 = activities.

^3 ^QLQ-C30 sum scores: ql = quality of life; pf = physical functioning; rf = role functioning; ef = emotional functioning; cf = cognitive functioning; sf = social functioning; fa = fatigue; nv = nausea & vomiting; pa = pain; dysp = dyspnea; sleep = sleeping; eat = eating; obst = obstipation; diarr = diarrhea; finan = financial difficulties.

_4 _QLQ-C30 items as dummy variables: 1 to 5 = dichotomous items; 6 to 27 = four level items.

* Not tested on external data-set.

All models used in this study were thus taken from other studies. Despite their use to investigate our methodological point, generalizability of mapping functions between different types of cancer or arthritis is an empirical matter that still needs thorough investigation.

### Analysis

First we determine if the mapping algorithms estimated on a relatively healthy patient sample overestimate utility values of patients in poor health. As the EQ-5 D is absent by definition, we need to specify a threshold value on the condition specific measure for which we would expect a regular mapping algorithm to overpredict utility values to be able to anticipate whether a mapping algorithm is expected to be inaccurate in a certain population. Then we develop a mapping algorithm for that population. Six steps are described below, aimed at systematically exploring the topic.

Step one. Each published algorithm used here was found in its original article to be successful at predicting mean EQ-5 D values. The same diagnostics have also been applied to the test model and indicate this model is successful at predicting mean EQ-5 D values. However, a successful prediction of a mean EQ-5 D utility value in a sample with a relatively high mean value does not guarantee a successful prediction in a sample with a much lower mean EQ-5 D value. Therefore we compare the predicted values are compared to the observed values over the range of observed EQ-5 D values.

Step two. It has been suggested that reporting a level '3' answer on EQ-5 D and the large utility decrement associated with it in the EQ-5 D country tariff is a cause of overprediction [3]. Using the UK tariff [14] an EQ-5 D utility value of .52 is the lowest obtainable value without a level 3 answer (state 22222), and 0.56 is the highest obtainable value with a level 3 answer (state 11311), which is respectively 0.57 and 0.64 for the Dutch tariff. These values will be used to interpret the distribution of utility values in the three samples.

Step three. The frequently observed bimodal distribution of utility values in patient samples has been associated with 'N3-term' [9] and the bimodal pattern has been presented by others as a specific feature of the EQ-5 D [21]. The N3 term is a model feature of the UK and Dutch EQ-5 D country tariff and adds an extra utility decrement if any dimension on the EQ-5 D scores a '3', representing extreme problems. However, it is hypothesized here that the 'N3' in itself does not cause a bimodal distribution. To test this, a random set of EQ-5 D cases is generated (N = 300) with an equal distribution of answer categories across the 5 domains.

Step four. Step one and two investigate whether the utility values of patients who report 'extreme problems' on at least one of the EQ-5 D dimensions are overpredicted. The next step is to investigate which QLQ-C30 and HAQ value is associated with level '3' answers on the EQ-5 D. The use of this exercise is to identify scores on the condition specific measure that are related to a possible cause of overprediction in mapped utility values: at those scores standard mapping algorithms might be inaccurate. As the QLQ-C30 provides no overall score, the functioning scale scores are used, since these have the highest correlation with EQ-5 D scores [22]. For the HAQ, the HAQ-DI value (which combines all items) is used.

Step five. The next step is exploring the performance of a separate algorithm for use on patients in poor health. An alternative algorithm will be developed on a sample in poor health, in this case on a within sample selection of patients which are in poor health as determined by the cut-off point identified in step 4. The utility value of the EQ-5 D, using the UK tariff will be regressed on the disease specific questionnaires. In the cancer population the algorithm will be developed on the multiple myeloma sample and tested on the non-Hodgkin's sample. A variety of model specifications are estimated using OLS. All algorithms are applied at the individual level. Mean utility values are used to compare predicted and observed values.

**Figure 1:**
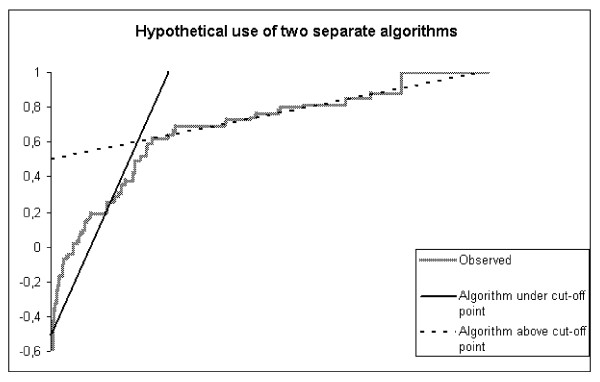
**Hypothetical use of two separate algorithms**.

Step six. Typically mapping algorithms are used to predict the mean utility value of a population that is in moderate to good health. In step 5 we specify a separate algorithm for patients in poor health which may reduce overprediction of utility values for patients in poor health. If only a part of the patient population is in poor health, a second algorithm is needed to be able to estimate the mean utility value of the entire sample. Thus computing utilities with the 'low utility' algorithm and a separate algorithm for patients in relatively good health may reduce prediction errors for a 'typical' sample where the majority of respondents are in moderate to good health. Such an approach would require two algorithms: one for the part of the population which is in poor health, as determined by a score under a cut-off point on the condition specific measure, and one for the population in better health, determined by a score higher than the cut-off point specified under step 4, as sketched in Figure 1. The 'low utility' algorithm estimated in step five will be complemented by a 'high' utility algorithm and tested on the non-Hodgkin's sample.

## Results

**Figure 2:**
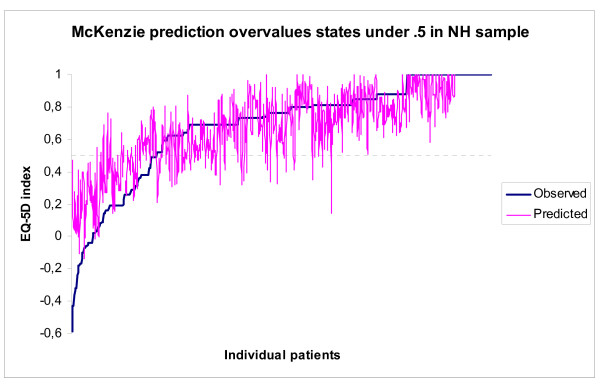
**McKenzie prediction overvalues states under 0**.5 in NH sample.

**Figure 3:**
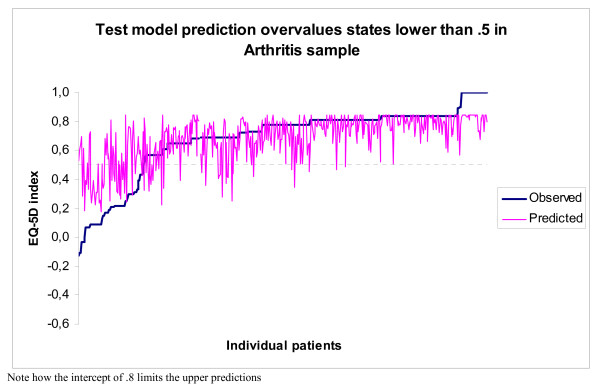
**Test model prediction overvalues states under 0**.5 in arthritis sample.

All mapping algorithms applied here suffer from overprediction at the lower end of the scale, where predicted values are higher than observed values for observed EQ-5 D utility values below ≈.5. Figure 2 and 3 compare predicted and observed EQ-5 D utility values, and are representative for the other mapping algorithms investigated in this study.

Step one. Figure 2 and 3 indicate that overprediction begins to occur around EQ-5 D utility value ≈.5. As is mentioned in the method section: the utility value of ≈.5 is related to the scoring 'extreme problems' on any EQ-5 D dimension. Patients that have one or more dimensions at level 3 have a maximum observed EQ-5D_UK tariff _score of 0.56 in the MM and NH samples and of 0.43 in the Arthritis sample. Patients that have no dimensions at level '3' have a minimum observed EQ-5D_UK tariff _score of 0.52 in all samples (state 22222). A utility value of 0.52 and lower guarantees the presence of at least one level 3 answer in the UK tariff. Scores higher than 0.52 but below 0.57 do not guarantee the absence of at least one level 3 answer. Interestingly enough Figure 3 shows overprediction to occur at a slightly higher value, but not at the expected 11311 score with utility value 0.64. Upon inspection the highest observed Dutch utility value for a state with a '3' is 0.55, for state 11321, thus the graph shows overprediction to start at that state.

**Figure 4:**
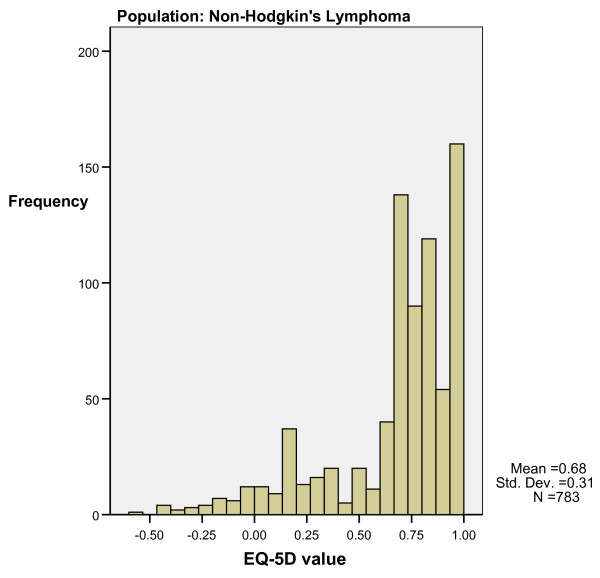
**Bimodal distribution of utility values in cancer population**.

**Figure 5:**
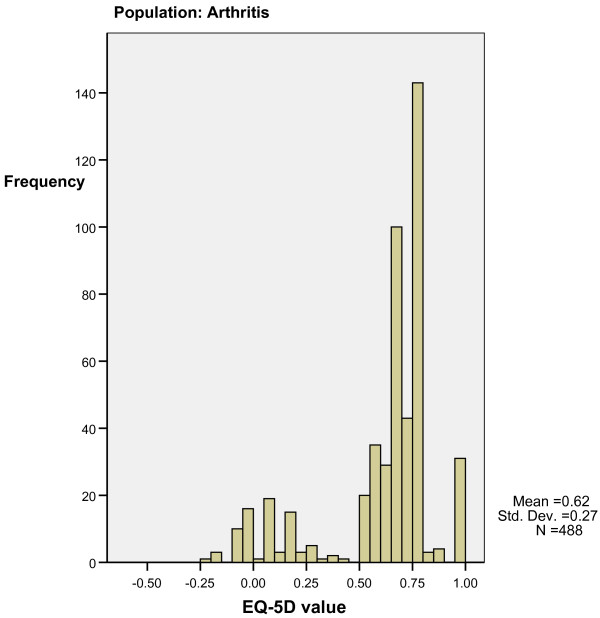
**Bimodal distribution of utility values in arthritis population**.

Step two. Minimum and maximum EQ-5 D scores of patients with or without at least one dimension at level 3 on the EQ-5 D inform our interpretation of Figure 4 and 5, which indicate bimodal distributions for MM and NH samples. A patient with a 'level 3' answer on EQ-5 D belongs to the left side 'poor health' distribution with a lower mean and less frequent observations than a patient without a 'level 3' answer. The area around a utility value of .5 can fall under either distribution, as indicated by the overlap in minimum and maximum values for cases with and without level 3 answers mentioned in step one.

**Figure 6:**
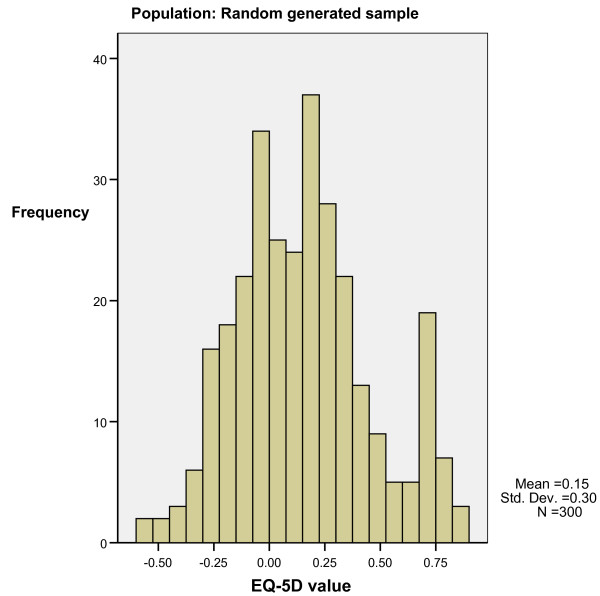
**Normal distribution of utility values despite 'N3-decrement'**.

Step three. Figure 6 shows the distribution of utility values for the randomly generated sample. The utility values have a normal distribution, suggesting that the bimodal distribution is not solely caused by the 'N3' term. The random sample (N = 300) had 163 unique health states. The 34 most frequent health states account for 36% of the observations, which is in stark contrast to the other samples. The NH sample (pooled N = 783) had 78 unique health states of which six states accounted for 53.5% of all observations. The MM sample (pooled N = 716) had 59 unique states of which seven states accounted for 62.1% of observations. The Arthritis sample (N = 488) had 49 unique states of which seven states accounted for 64% of the data. The combination of the EQ-5 D country tariff and distribution of responses across severity levels seem to be the cause of the bimodal distribution of EQ-5 D utility values. Few people have level '3' answers, many have level 1 or 2 answers and only a small amount of states cover most of the observations.

**Figure 7:**
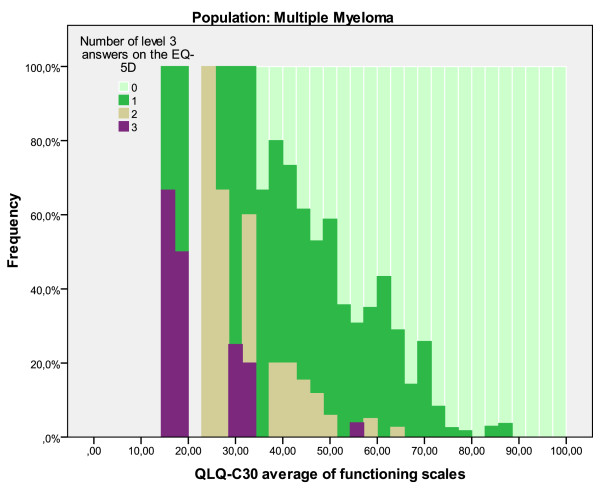
**Number of level 3 answers on EQ-5D can inform decision on appropriateness of mapping function**.

Step four. Mapping algorithms overpredict utility values under 0.5, which are for patients with 'extreme problems' on at least one of the five EQ-5 D dimensions. This means that mapped utility values are inaccurate for those patients with scores on the condition-specific measure that are associated with an EQ-5 D utility value below 0.5. However, scores on the HAQ and QLQ-C30 do not provide a straightforward indication of the accuracy of the use of a mapping algorithm. For example, a patient average on the QLQ-C30 functioning scales of 70 could belong to an EQ-5 D utility value between as low as .21 or as high as 1. However, Figure 7 shows that at least half of the patients with an average value of the QLQ-C30 functioning scale lower than 55 have level 3 answers on the EQ-5 D. Although it is a somewhat arbitrary cut-off point, an average of 45 on the functioning scales is a clear indication of the expected overprediction of a mapping algorithm, for at that value approximately 86% of patients in these samples have at least one level 3 response.

**Figure 8:**
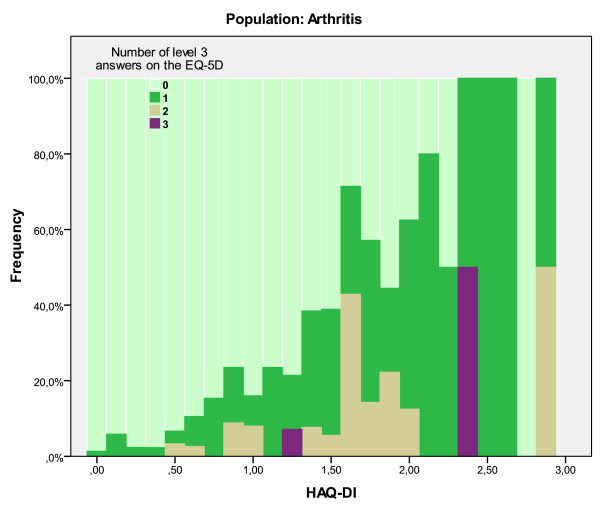
**Number of level 3 answers on EQ-5 D can inform decision on appropriateness of mapping function**.

The HAQ-DI values faced similar problems: a HAQ-DI value of 1.5 (moderate to severe disability) can be associated with an EQ-5 D utility value as low as .21 to .3 or as high as .71 to .8. Figure 8 does indicate that at HAQ-DI values <1.6, over 50% of patients have at least one level 3 response on the EQ-5 D. A HAQ-DI > 2.0 is a clear indication of the expected overprediction of a regular mapping algorithm, for at that value, approximately 72% of patients in this sample has at least one level 3 response.

Step five. The within sample population of cases in poor health (QLQ-C30 <45, HAQ-DI > 2.0) was relatively small (N = 18 Arthritis sample, N = 25 at t = 0 NH-sample, N = 40 at t = 0 MM-sample). Within those subsamples, EQ-5 D was regressed on QLQ-C30 and HAQ using a variety of regression model specifications. The mapping model was developed on the MM-sample, and tested on the NH-sample. The QLQ mapping algorithm contained 5 items after backwise selection, and included items as categorical variables. The mapping algorithm was applied on the NH sample for patients with QLQ average on the functioning scales < 45. In comparison to the standard mapping algorithms, the utility model for patients in poor health outperforms the model from the literature (Table 3) for this selection of the sample and reduces root mean square error by .06 in the first 4 timepoints. As can be seen from the maximum score, 1 individual did not seem to have filled in the EQ-5 D correctly and had a utility value of 1 (but a low score of 25 on the EQ-5 D visual analogue scale). A similar pattern was observed for the last four timepoints, but not deemed trustworthy due to small sample size (N < 8 for the last 4 timepoints of the QLQ-C30 follow up data). The predicted values showed less prediction error than the standard mapping algorithms, but still did not accurately predict mean utility samples in this selection of the sample with root mean squared error of 0.18.

**Table 3:** Predicted and observed values in N-H population with QLQ-C30 < 45

Timepoint		N	Minimum	Maximum	Mean	Std. Deviation
Baseline	Observed EQ-5D	25	-,36	1,00	**,18**	,39
	Predicted McKenzie & Van der Pol	24	-,14	,56	**,25**	,15
	Predicted 'low'	25	-,34	,54	**,14**	,22
		
T = 1	Observed EQ-5D	17	-,43	,64	**,16**	,26
	Predicted McKenzie & Van der Pol	17	-,01	,62	**,32**	,17
	Predicted 'low'	17	-,07	,29	**,14**	,11
		
T = 2	Observed EQ-5D	16	-,33	,38	**,10**	,18
	Predicted McKenzie & Van der Pol	16	,16	,56	**,32**	,12
	Predicted 'low'	16	-,03	,41	**,16**	,11
		
T = 3	Observed EQ-5D	13	-,24	,31	**,07**	,17
	Predicted McKenzie & Van der Pol	13	-,01	,55	**,31**	,14
	Predicted 'low'	13	-,17	,42	**,18**	,17

For the REACH study, only a development dataset was available but for both cut-off points (HAQ-DI > 1.6 and HAQ-DI >2.0) the regression model was underpowered with no significant predictor variables due to the small sample size and low correlations between HAQ sum scores and EQ-5 D utilities. Step six was performed with QLQ-C30 models only.

Step six. The 'high' and 'low' utility algorithms, predicting UK EQ-5 D utilities are presented in Table 4. The low utility model in step five was supplemented with a high utility model developed on patients with an average sum score on the functioning scales of the QLQ-C30 >45. Application of the algorithm in the non-Hodgkin's sample was similar to the development: patients who were in poor health got assigned the utility value as predicted from the 'low utility' model and the rest got assigned the utility value as predicted from the 'high utility' model. The combined variable of predicted values had a lower root mean square error (0.02 lower on average) and a larger range of predicted values than the other QLQ-C30 models discussed in this paper. This suggests a modest improvement and indeed led to a slightly better estimate of the mean utility values (Table 5). Due to data restrictions like few observations of poor health states and the model specifications (items treated as categorical variables) the uncertainty around the parameter estimates of the low utility model was almost three times higher than the uncertainty around the parameter estimates of the high utility model.

**Table 4:** Coefficients of the separate utility algorithms

		Unstandardized Coefficients		*p*
			
Model: Low utilities		**Coeff**.	Std. Error	
Items	(Constant)	0,773	0,13	0,00
	3	-0,117	0,07	0,01
	4	-0,244	0,07	0,00
	5	-0,124	0,07	0,08
	9_dummy1	-0,135	0,09	0,14
	9_dummy2	-0,053	0,10	0,60
	9_dummy3	-0,274	0,12	0,02
	21_dummy1	-0,181	0,09	0,05
	21_dummy2	-0,144	0,09	0,13
	21_dummy3	-0,358	0,15	0,02
Model: high utilities		Unstandardized Coefficients		*p*
			
		Coeff	Std. Error	
Items	(Constant)	0,970	0,01	0,00
	1	-0,065	0,02	0,00
	2	-0,050	0,01	0,00
	3	-0,072	0,02	0,00
	4	-0,028	0,02	0,16
	5	-0,199	0,03	0,00
	9_dummy1	-0,080	0,02	0,00
	9_dummy2	-0,095	0,02	0,00
	9_dummy3	-0,233	0,05	0,00
	11_dummy1	0,001	0,01	0,94
	11_dummy2	-0,015	0,02	0,46
	11_dummy3	-0,019	0,03	0,56
	15_dummy1	-0,027	0,02	0,22
	15_dummy2	-0,158	0,05	0,00
	15_dummy3	-0,070	0,12	0,57
	19_dummy1	-0,029	0,02	0,05
	19_dummy2	-0,073	0,02	0,00
	19_dummy3	-0,167	0,05	0,00
	23_dummy1	-0,028	0,01	0,02
	23_dummy2	-0,062	0,03	0,03
	23_dummy3	-0,563	0,13	0,00
	27_dummy1	-0,055	0,01	0,00
	27_dummy2	-0,164	0,02	0,00
	27_dummy3	-0,248	0,04	0,00

**Table 5:** Comparison of algorithms in N-H population

Time point		N	Minimum	Maximum	Mean	Std. Deviation
Baseline	Observed EQ-5D	117	-,36	1,00	**,60**	,37
	Predicted McKenzie & Van der Pol	106	-,14	1,06	**,61**	,27
	Predicted Combined	108	-,34	,97	**,60**	,32
		
T = 1	Observed EQ-5D	124	-,43	1,00	**,64**	,33
	Predicted McKenzie & Van der Pol	115	-,01	1,03	**,66**	,24
	Predicted Combined	120	-,07	,97	**,63**	,25
		
T = 2	Observed EQ-5D	116	-,33	1,00	**,67**	,30
	Predicted McKenzie & Van der Pol	111	,16	1,03	**,66**	,21
	Predicted Combined	111	-,03	,97	**,66**	,25
		
T = 3	Observed EQ-5D	103	-,24	1,00	**,65**	,31
	Predicted McKenzie & Van der Pol	96	-,01	1,03	**,62**	,23
	Predicted Combined	99	-,17	,97	**,63**	,25
		
T = 4	Observed EQ-5D	101	-,43	1,00	**,72**	,32
	Predicted McKenzie & Van der Pol	94	,03	1,05	**,73**	,23
	Predicted Combined	95	-,17	,98	**,71**	,24
		
T = 5	Observed EQ-5D	87	-,18	1,00	**,75**	,24
	Predicted McKenzie & Van der Pol	82	-,11	1,05	**,75**	,23
	Predicted Combined	84	-,13	,98	**,74**	,21
		
T = 6	Observed EQ-5D	76	-,59	1,00	**,73**	,32
	Predicted McKenzie & Van der Pol	68	,05	1,06	**,77**	,23
	Predicted Combined	71	-,13	,97	**,76**	,22
		
T = 7	Observed EQ-5D	59	,06	1,00	**,77**	,21
	Predicted McKenzie & Van der Pol	59	,00	1,04	**,78**	,22
	Predicted Combined	59	-,13	,98	**,78**	,20

## Discussion

This paper explored causes of EQ-5 D utility values for patients in poor health when mapping from a non-preference-based measure, and investigated a possible solution to the problem. We examined the association between the cause of the overestimation and values on the condition specific questionnaire at which overprediction occurs. Our findings suggest that the main cause of overestimation is a combination of the large decrement in utility values in the UK and Dutch EQ-5 D tariffs for having one or more dimensions at level '3', along with few observed responses at level '3'. We argue that this, alongside the large number of EQ-5 D responses at the least severe level, leads to a bimodal distribution of the utility data. A result is that the most linear prediction models can not adequately describe low utility values. We found that the values on the condition specific questionnaire can help inform decisions about the expected errors and hence accuracy of standard mapping algorithms, and that the use of a separate mapping algorithm specified for patients in poor health reduces the amount of overprediction for these patients. Combining such a function with a 'high utility' algorithm leads to a modest improvement of predictions.

Our findings, in accordance with the literature, suggest that the ≈.5 value of the EQ-5D_UK tariff _is the point at which mapping algorithms start to overpredict utility values. The reason it is the ≈.5 is due to the fact that values under ≈.5 belong to patients who have extreme problems on at least one dimension of EQ-5 D. As the purpose of mapping algorithms is to predict EQ-5 D values when EQ-5 D was not included in the trial, such a value is not informative for the application of mapping algorithms. Here we explored the use of condition specific measures (that we are mapping from) to indicate the expected accuracy of a standard mapping algorithm. An alternative mapping algorithm can then be developed for use in patients in poor health. We found that the ≈.5 utility value itself is not a very useful measure of association with QLQ-C30 or HAQ-DI values, since there is not a one-to-one relationship between measures meaning that a large range of QLQ-C30 and HAQ scores are associated with the ≈.5 EQ-5 D value. Since scoring a '3' on the descriptive system of EQ-5 D is related to the problem of overprediction, we took an alternative approach using the scores on the condition-specific measure that correspond to having at least one level '3' response. Below a QLQ-C30 average of the functioning scale of 55, about half of the patients scores level 3 answers on the EQ-5 D, as do patients with HAQ-DI > 1.6. At these scores, standard mapping algorithms are likely to overpredict utility values. More conservative and somewhat arbitrary cut-off values we determined are > 2.0 for HAQ-DI and < 45 for the average of the QLQ-C30 functional scales. These cut-off points represent very severe health problems: 45 for the QLQ-C30 is associated with severe cases like post-radiotherapy patients with metastatic and/or cardio-respiratory disease [23]; a HAQ-DI value under 2.0 represents severe to very severe RA [18]. At these more conservative values, a standard mapping algorithm is likely to be inaccurate.

A separate utility mapping algorithm estimated on a sample with poor health status is far better at predicting utility values for patients in poor health, when it is possible to estimate such a function. However, using categorical variables introduced problems with perfect colinearity in the low utility model, and the HAQ sample did not allow the estimation of a low utility model due to poorer correlation with EQ-5 D and smaller sample size than QLQ-C30. A model based on sum scores did not suffer from these restrictions but introduced larger prediction errors. The result is a model for low utilities that only uses 5 items of the QLQ-C30 as predictor variables. Item 3 (trouble taking a short walk), 4 (need to stay in bed or a chair), 5 (need help with eating, dressing, washing or using the toilet) 9 (pain) and 21 (feeling tense) together represent physical functioning, emotional functioning and pain. Consequently other quality of life drivers such as role functioning or fatigue are not represented which may lead to problems when applying the function in other cancer types. Furthermore, OLS models used in all mapping algorithms reported here are more precise around mean values than for extremes, which results also in underprediction for utility values near to 1, most notably when regressing EQ-5 D on HAQ. Thus estimating and applying mapping algorithms on datasets with large deviations in health status is likely to be problematic. The extent to which a deviation can be considered 'large' is difficult to assess, since it depends on how a change on the scale of the questionnaire relates to a change on the EQ-5 D index values.

Cut-off points like the ones specified in this study can be used to inform whether a regular mapping algorithm from the literature would suffice or whether a 'low utility algorithm' is better at assessing the quality of life for those patients. Cut-off points can indicate whether there are patients in poor health and therefore whether predicted utility values are likely to suffer from overprediction if only a standard mapping algorithm has been used. Cut-off points can therefore inform users and policy makers whether mapped estimates should be treated with great caution. A weakness of the approach may be that there is no clear cut relation between the break point of utility values in the distribution and values on the condition specific measures. Besides, prediction errors might be reduced even more if there were several mapping functions for each 'severity group'. However, the relation between the condition specific measure and the preference-based measure may not be clear cut enough to identify more sub-groups.

Although overprediction proved to be less of a problem for patients in poor health with our combined prediction model, the largest part of the sample is not in very poor health. This explains why predictions of the mean, as presented in Table 5 do not show much improvement compared to the McKenzie model. However, predicted EQ-5 D values do not capture the full range of observed EQ-5 D values due to overprediction. As a consequence, they have 'tighter' confidence intervals around the QALY estimates as presented in Table 6 (survival is hypothetical). In probabilistic sensitivity analysis this results in less uncertainty around the estimate of cost per QALY, but that is an incorrect representation of reality.

**Table 6:** Hypothetical QALY confidence intervals

**T = 0**	**Utility (SD)**	**Survival^1^**	**QALY (SD)**	**QALY 95%CI**
	
Observed	0,60 (0,37)	5	2,97 (1,8)	2,64 - 3,31
McKenzie	0,61 (0,27)	5	3,06 (1,3)	2,80 - 3,32
Predicted Combined	0,60 (0,32)	5	2,97 (1,6)	2,67 - 3,28

^1 Hypothetical figure^

In addition to the tighter confidence intervals, using mapped utility values may result in an underestimation of the utility-gain between time intervals. As the utility values of patients in poor health are systematically overpredicted, individuals who in reality would improve from poor health to better health (i.e. from a value <0.5 to a value >0.5) would have an underestimated utility gain when using mapped EQ-5 D utilities.

A main point of concern in any effort to map onto a preference-based questionnaire is generalizability of the results. As mentioned earlier, it must be stressed that although the cut-off points presented here are empirically supported by our study, they cannot be considered transferable or generalizable to other types of cancer or arthritis samples prior to thorough empirical testing in different datasets.

The issue of generalizability also applies to the presented methodology. This study focussed on mapping onto EQ-5 D for patients in poor health. The methodology proposed here only applies to mapping onto EQ-5 D using the UK or the Dutch country tariffs. We observed that individuals who report 'extreme problems' on one of the five EQ-5 D dimensions receive overestimated utility values from published mapping functions. Our suggestion is that this is caused by the large utility decrement applied to scoring 'extreme problems' in the UK and Dutch EQ-5 D country tariff, combined with only a few observations of 'extreme problems'. However, other EQ-5 D country tariffs may not have large utility decrements for all 'extreme problems' scores. For instance, the total decrement for scoring 13111 ('extreme problems' on the self-care dimension of EQ-5D) has a total utility decrement of 0.564 in the UK tariff and 0.254 in the Japanese tariff. These differences in preferences between populations may be of influence on the methodology used to identify the part of the population which is in poor health and where increased prediction errors are observed. However, if those patients can be identified, specifying a separate mapping function for that part of the populations is still a suggested option to reduce prediction error.

We also investigated the option of combining the application of a low and high utility models, to see if the improvement found for low utility values would contribute to a better estimate of mean EQ-5 D utility values in a sample where only a part of the patients is in poor health. The model led to a modest improvement in root mean square error and range of the values. The range of the values is important, as that allows more statistical sensitivity. Further research is needed to determine if specifying two functions and combining them is to be favoured over other approaches. For instance, the problem mentioned above about the limited number of items available due to collinearity may be solved by using a larger dataset which provides more accurate predictions for summed scores. The approach could also be undertaken using regression techniques such as the probit model and a two-part model and this is an area for future research. An obvious attempt would be to raise variables to a power to allow non-linearity, but a recent study still reported overprediction under a utility value of around .6 for a model with significant second order predictors [24]. Alternatively, stepped linear regression with a specified break-point may allow the utility function to 'curve' according to observed values, but specifying such a breakpoint is not clear cut as is shown in this study.

## Conclusion

As the use of mapping in cost-effectiveness analyses of medical interventions is becoming more frequent, guidelines on the appropriateness of using mapping and specific mapping algorithms are needed. We investigated the often observed problem of overprediction in mapping and analysed the use of cut-off scores for the condition specific measures QLQ-C30 and HAQ-DI to indicate when the use of a separate mapping algorithm for patients in poor health is the favoured approach. Overprediction of utility values for patients in poor health can be greatly reduced by predicting the utility values of these patients using a separate mapping algorithm specified and estimated specifically for these patients, when deemed necessary.

## Competing interests

The authors declare that they have no competing interests.

## Authors' contributions

MV carried out statistical analysis and drafted the manuscript. DR, JB and ES have been involved in interpreting the data and helped to draft the manuscript. All authors read and approved the final manuscript.
